# Antimicrobial Resistance, Class 1 Integrons, and Genomic Island 1 in *Salmonella* Isolates from Vietnam

**DOI:** 10.1371/journal.pone.0009440

**Published:** 2010-02-26

**Authors:** An T. T. Vo, Engeline van Duijkeren, Wim Gaastra, Ad C. Fluit

**Affiliations:** 1 Department of Infectious Diseases and Immunology, Faculty of Veterinary Medicine, Utrecht University, Utrecht, The Netherlands; 2 Department of Medical Microbiology, University Medical Centre Utrecht, Utrecht, The Netherlands; 3 Faculty of Animal Husbandry and Veterinary Medicine, Nong Lam University, Ho Chi Min City, Vietnam; Charité-Universitätsmedizin Berlin, Germany

## Abstract

**Background:**

The objective was to investigate the phenotypic and genotypic resistance and the horizontal transfer of resistance determinants from *Salmonella* isolates from humans and animals in Vietnam.

**Methodology/Principal Findings:**

The susceptibility of 297 epidemiologically unrelated non-typhoid *Salmonella* isolates was investigated by disk diffusion assay. The isolates were screened for the presence of class 1 integrons and *Salmonella* genomic island 1 by PCR. The potential for the transfer of resistance determinants was investigated by conjugation experiments. Resistance to gentamicin, kanamycin, chloramphenicol, streptomycin, trimethoprim, ampicillin, nalidixic acid, sulphonamides, and tetracycline was found in 13 to 50% of the isolates. Nine distinct integron types were detected in 28% of the isolates belonging to 11 *Salmonella* serovars including S. Tallahassee. Gene cassettes identified were *aadA1*, *aadA2*, *aadA5*, *bla*_PSE-1_, *bla*_OXA-30_, *dfrA1*, *dfrA12*, *dfrA17*, and *sat*, as well as open reading frames with unknown functions. Most integrons were located on conjugative plasmids, which can transfer their antimicrobial resistance determinants to *Escherichia coli* or *Salmonella* Enteritidis, or with *Salmonella* Genomic Island 1 or its variants. The resistance gene cluster in serovar Emek identified by PCR mapping and nucleotide sequencing contained SGI1-J3 which is integrated in SGI1 at another position than the majority of SGI1. This is the second report on the insertion of SGI1 at this position. High-level resistance to fluoroquinolones was found in 3 multiresistant *S.* Typhimurium isolates and was associated with mutations in the *gyrA* gene leading to the amino acid changes Ser83Phe and Asp87Asn.

**Conclusions:**

Resistance was common among Vietnamese *Salmonella* isolates from different sources. Legislation to enforce a more prudent use of antibiotics in both human and veterinary medicine should be implemented by the authorities in Vietnam.

## Introduction

Non-typhoid *Salmonella* infection is one of the main zoonotic diseases in developed [1], [2] and developing countries [3], [4]. The ease with which people can travel between distant countries and the exchange of food between countries by global trade has contributed significantly to the spread of food-borne diseases. Multidrug-resistant (MDR) *Salmonella* isolates are a direct threat to human health when this multidrug resistance interferes with treatment and an indirect threat when resistance can be transferred to other human pathogens [5]. Therefore, antimicrobial susceptibility monitoring is important for the detection of resistant clinical isolates and for the surveillance of antimicrobial resistance.

A strong relationship between MDR *Salmonella* strains and the presence of integrons has been proven [6], [7]. Class 1 integrons are the most common integron type present in clinical isolates of the Enterobacteriaceae. Class 1 integrons, and transferable elements like conjugative plasmids or transposons, play an important role in the carriage and dissemination of antimicrobial resistance genes due to their ability to incorporate or excise one or more resistance gene cassettes [8], [9]. Antibiotic resistance gene clusters in class 1 integrons located on the chromosomal *Salmonella* Genomic Island 1 (SGI1) have been demonstrated in *S.* Typhimurium DT104 [10]. The integron is located between genes S027 and S044 of SGI1 [11], [12], [13], [14], [15]. Recently, the integration of a complex integron in gene S023 of the SGI1 was reported [16], [17]. The SGI1-associated MDR region consists of a complex integron carrying the *aadA2*, *bla*_PSE_, *floR*, *tetR*, and *tet*(G) genes. In several *Salmonella* serovars, including strains of *S.* Typhimurium DT104, a number of SGI1 variants (SGI1-A to J) have been detected. SGI1 is transmissible, but only in the presence of a helper plasmid. This mobility of SGI1 by conjugative mobilization may contribute to the spread of antibiotic resistance genes between different *S. enterica* serovars and between *Salmonella* and other bacterial pathogens [18].

The aims of this study were to investigate (i) the antimicrobial resistance of Vietnamese *Salmonella* isolates collected from humans, livestock and meat (ii) the prevalence and characteristics of class 1 integrons in these isolates and (iii) the resistance gene clusters present in SGI1.

## Results

### Resistance Phenotype

Onehundred-and-ten (37%) Vietnamese *Salmonella* isolates were fully susceptible to all 15 antimicrobials tested (Table 1). No ceftazidime-resistant isolate was found. Nearly two thirds of the collection (187 isolates) showed resistance to at least one antimicrobial agent. More than 40% (n = 125) of the isolates belonging to 17 serovars were resistant against ≥2 antimicrobials. Resistance to six or more antimicrobials was found in 51 isolates (17%). Resistance to gentamicin was found among human (14%) and porcine (20%) salmonellae, especially *S.* Typhimurium isolates. Of the poultry isolates, 80% were resistant against nalidixic acid. Three norfloxacin-resistant *S.* Typhimurium isolates were found and all were isolates from humans (n = 3).

**Table 1:** Number of resistant *Salmonella* isolates belonging to different serovars isolated from humans, cattle, pigs and poultry in Vietnam by an agar diffusion method*.

Sources/serovars	A	Ac	Ce	Cf	S	G	K	C	Na	No	Ci	T	Su	Tp	Co	MDR isolates N (%)
**Human (56)**	**24**	**2**	**2**		**23**	**14**	**11**	**13**	**21**	**2**	**2**	**30**	**28**	**18**		**28 (50)**
Typhimurium (21)	14	2	1		14	11	10	6	11	2	2	15	15	12		
Enteritidis (7)	4				4							4	4			
Emek (2)								2	2				2	2		
Others (26)	6		1		5	3	1	5	8			11	7	4		
**Cattle (63)**	**18**	**2**	**1**		**6**	**2**	**2**	**3**	**10**	**1**	**1**	**19**	**7**	**4**		**12 (19)**
Anatum (15)	12		1		2			1	9			14	2	2		
Typhimurium (3)	3	1			3	2	2	2	1	1	1	3	3	2		
Others (45)	3	1			1							2	2			
**Pig (111)**	**35**				**25**	**20**	**21**	**8**	**6**			**69**	**26**	**24**		**34 (30.6)**
Anatum (29)	13				3			2	4			24	2	1		
Typhimurium (23)	18				18	18	18	2				18	18	18		
Derby (13)	2				2	2	2	1	2			13	2	2		
Others (46)	2				2		1	3				14	4	3		
**Poultry (67)**	**9**	**2**	**3**		**23**	**2**	**14**	**34**	**54**			**30**	**40**	**35**	**1**	**51 (76.1)**
Emek (26)	1	1	1			2		28	26			1	28	28		
Blockley (14)					14		14	1	18			14	1	1		
Albany (3)	3				1			3	3			2	3	3	1	
Others (24)	5	1	2		8			2	7			13	8	3		
TOTAL (267)	86	6	6		77	38	48	58	91	3	3	148	101	81	1	125 (42)
% resistant**	29	2.0	2	0	26	13	16	20	31	1	1.0	50	34	27	0.3	
% intermediate	1	8	2	0	19	0	0.3	5	8	0	0.6	9	0	0	0	
% susceptible	70	90	96	100	55	87	83	75	61	99	98.4	41	66	73	99.7	

Abbreviations used: N, number of the isolates tested A, ampicillin (10µg); Ac, amoxicillin/clavulanic acid (30/15µg); Ce, cephalothin (30µg); Cf, ceftazidime (30µg); S, streptomycin (10µg); G, gentamicin (10µg); K, kanamycin (30µg); C, chloramphenicol (30µg), Na, nalidixic acid (30µg); No, norfloxacin (10µg); Ci, ciprofloxacin (5µg); T, tetracycline (30µg); Su, sulphonamide (300µg); Tp, trimethoprim (5µg), Co, colistin (10µg); MDR, multidrug-resistant.

* The number of isolates resistant to a particular antimicrobial agent is given below each antimicrobial.

** The percentage of the total number of isolates resistant, intermediate resistant or susceptible for a particular antimicrobial is indicated in the last three rows below each antimicrobial.

### Integrons and Gene Cassettes

The prevalence of class 1 integrons was high (28%). Nine different profiles of class 1 integrons (Table 2) were detected in 83 isolates belonging to 11 serovars. The gene cassettes found in these integrons included the *aadA1*, *aadA2* and *aadA5* genes encoding resistance to streptomycin and spectinomycin, the *bla*_PSE-1_ and *bla*_OXA-30_ genes conferring resistance to β-lactams, the *dfrA1*, *dfrA12* and *dfrA17* genes encoding resistance against trimethoprim, the *sat* gene mediating streptothricin resistance and open reading frames encoding proteins of an unknown function. Phenotypic resistance to a certain antimicrobial drug was observed in all isolates carrying the corresponding gene cassettes. The transfer of the integrons and the antimicrobial resistance determinants (AAcGKSTSuTp, CSTSuTp, and CSSu) to *E. coli* was possible from 17 of the 83 integron-positive isolates of serovar Typhimurium, Anatum, and Agona, respectively (Table 3). Ten *S.* Typhimurium isolates from 17 isolates tested could transfer their integrons and resistance determinants to *S.* Enteritidis. This was demonstrated by the fact that the *E. coli* and *S.* Enteriditis transconjugants were *int* positive and obtained the phenotypic resistance patterns of the donors.

**Table 2:** Characterization of class 1 integrons of *Salmonella* isolates from human and animal origin in Vietnam.

IP^a^	Size in bp (isolate ID)	RE^b^ 1	Fragments (bp)	RE 2	Fragments (bp)	Gene cassette	Accession number
I	1010 (V237)	*EcoR*I	561; 449	*Hpa*II	411; 246; 138; 73; 61; 57; 24	*aadA2*	DQ238100
	1197	*Hinc*II	703; 351; 143	*Hpa*II	826; 371	*bla*_PSE-1_	DQ238099
II	1242 (V84)					*dfrA1,orfC*	DQ238102
	1198					*bla*_PSE-1_	DQ238101
V	1010 (V171)	*EcoR*I	561; 449	*Hpa*II	411; 246; 138; 73; 61; 57; 24	*aadA2*	DQ238098
X	-						
XI	1242 (V14)	Hinc*II*	656; 490; 96	*Hpa*II	762; 480	*dfrA1, orfC*	DQ238097
XII	1914 (V80)	Hinc*II*	1303; 611	Hpa*II*	538; 464; 246; 196; 138; 116; 73; 57; 24	*aadA2, orfF, dfrA12*	DQ238105
XIII	1700 (V57)					*dfrA17, aadA5*	
	1914					*aadA2, orfF, dfrA12*	
	2010					*bla*_OXA-30_, *aadA1*	DQ861642
XIV	1914 (V58)					*aadA2, orfF, dfrA12*	
	2010					*bla*_OXA-30_, *aadA1*	
XV	627 (V48)	Bcl*I*	480; 147	Nci*I*	351; 151; 125	*sat*_(partial)_	DQ284538

a integron profile nomenclature followed that from a previous study [41]. Profiles (XI–XV) are designated in this study.

b RE: restriction endonuclease.

- no product obtained in CS-PCR or inverted PCR.

**Table 3:** Antimicrobial resistance characteristics of MDR *Salmonella* isolates from human and animal origin in Vietnam.

Multidrug-resistance patterns	Serovars (animal/human isolates)	IP types	Conjugation		SGI 1	
			*Ec*	*S*E	Type	Excision
SSu	Derby (2/0)	V(2)^a^	-	nt	SGI1-C	nt
ATNa	Anatum (13/0)	-	nt	nt	nt	nt
CSSu	Agona (1/0)	X (1)	+ (1)	-	-	nt
STSu	London (3/0)	-	nt	nt	nt	nt
ACTNa	Anatum (1/0)	-	nt	nt	nt	nt
ASSuG	Kedougou (0/1)	XV (1)	-	nt	-	nt
ASTSu	Enteritidis (0/4)	-	nt	nt	nt	nt
CSuTpNa	Emek (23/1)	XI (24)	-	nt	SGI1-J3	-
CTSuTp	Panama (1/0)	-	nt	nt	nt	nt
STKNa	Blockley (13/0)	-	nt	nt	nt	nt
STSuNa	Tm pt 507 (0/1), Hadar (0/1)	-	nt	nt	nt	nt
ACSuTpNa	Albany (1/0)	II (1)	-	nt	SGI1-F	-
ACTSSu	Tm pt 506 (0/1)	I (1)	-	nt	SGI1	+
ACTSuTp	Panama (1/1)	X (2)	-	nt	-	nt
ATSuTpNa	Anatum (1/0)	X (1)	-	nt	-	nt
CSSuTpNa	Emek (0/1)	XI (1)	-	nt	SGI1-J3	-
CSuTpGNa	Emek (2/0)	XI (2)	-	nt	-	nt
CSTKNa	Blockley (0/1)	-		nt	nt	nt
CSTSuTp	Anatum (1/0)	XII (1)	+ (1)	nt	-	nt
ACSTSuG	Kedougou (0/1)	X (1)	-	nt	-	nt
ACSTSuNa	Tm RDNC (0/1), Tm pt 506 (1/0)	I (2)	-	nt	SGI1	+
ASTSuGNa	Tm RDNC (0/1)	I (1)	-	nt	SGÍ1	+
ASTSuTpG	Tm 90 (4/0)	XII (4)	+ (1)	+ (1)	-	nt
ASTSuTpNa	Schwarzengrund (1/0)	XII (1)	-	nt	-	nt
ACSTSuTpNa	Albany (1/1)	II (2)	-	nt	SGI1-F	-
ATSuTpAcCeNa	Schwarzengrund (1/0)	XII (1)	-	nt	-	nt
ACSTSuTpNa	Tallahassee (2/0)	II (2)	-	nt	SGI1-F	-
CSTSuTpKNa	Blockley (1/0)	-	nt	nt	nt	nt
ASTSuTpGK	Tm pt 90 (8/6), Tm pt 507 (2/0), Tm RDNC (1/0), Tm pt 510 (1/0)	XII (18)	+ (10)	+ (6)	-	nt
ASTSuTpGNa	S.*enterica* (I) 4, (5) 12∶1∶ - (0/1)	XII (1)	-	nt	-	nt
ASTSuTpGNa	Tm UT (1/0)	XII (1)	+ (1)	+ (1)	-	nt
ACSTSuTpCeNa	Anatum (1/0)	V (1)	-	nt	-	nt
ACTSuTpAcCeNa	Emek (1/0)	XI (1)	-	nt	SGI1-J3	-
ACSTSuTpCoNa	Albany (1/0)	II (1)	-	nt	SGI1-F	-
ASTSuTpGKNa	Tm 90 (1/1)	XII (2)	+ (1)	+ (1)	-	nt
ACSTSuTpGK	Derby (1/0)	XII (1)	-	nt	-	nt
ACSTSuTpGK	Tm 90 (2/0)	XII (2)	+ (1)	+ (1)	-	nt
ACSTSuTpGNa	Tm 90 (1/0)	-	nt	nt	nt	nt
ASTSuTpKGNa	Tm 90 (1/0), Tm RDNC (1/0)	-	nt	nt	nt	nt
ACSTSuTpAcGKNaNoCi	Tm 507 (0/1), Tm UT (0/1)	XIV (2)	+ (1)	-	-	nt
ACSTSuTpAcCeGKNaNoCi	Tm UT (0/1)	XIII (1)	-	nt	-	nt

Abbreviations used: A, ampicillin; C, chloramphenicol; S, streptomycin; T, tetracycline; Su, sulfonamides; Tp, trimethoprim, Ac, amoxicillin; Ce, cephalothin; G, gentamicin; K, kanamycin; Na, nalidixic acid; No, norfloxacin; Ci, ciprofloxacin; Co, colistin.

Tm, Typhimurium; RDNC, reaction does not conform to any recognized phage types; UT, untypeable phage; *Ec*, *E. coli* as the recipient; *SE*, *S.* Enteritidis as the recipient; QRDR, quinolone resistance determining region.

- not found; nt, not tested.

a number in brackets: number of isolate(s).

### *Salmonella* Genomic Island 1

In serovar Typhimurium, Derby, Albany, and Tallahassee, SGI1 and the variants SGI1-C and SGI1-F were found (Table 3). The three *S.* Emek isolates with the phenotypic resistance pattern CNaSSuTp, CNaTpSu, and AAcCeCNaTTpSu (V14, V28 and V116, respectively, were positive in the PCRs for SGI1 [14]. Remarkably, an integron structure was not present between SGI1 genes S027–S044, instead it was present in gene S023. PCRs specific for the genes commonly present in the integron structures of SGI1 showed the presence of a structure identical to SGI-J3 [16].

### Circular Form of SGI1

Excision and circularization of a SGI is the first step in horizontal transfer to other bacteria. To study the possible excision of SGI1 from the genome as a circular form, a PCR specific for the circular form of SGI1 was used. The SGI1 in 3 *S.* Typhimurium isolates proved to be present in its circular form. Nucleotide sequencing showed that the fragment of ca. 430 bp obtained by SGIc-PCR from a SGI1 carrying *S.* Typhimurium isolate (V54) was identical to the sequence of the *S004* gene, the right end of SGI1 up to the insertion site of the cryptic retronphage [18] in the sequence deposited in the GenBank under accession number AF261825.2. No PCR product indicating the presence of the circular form of SGI1 was obtained with DNA from other serovars.

### Fluoroquinolone Resistance

No *qnrA* gene was found in the three norfloxacin/ciprofloxacin-resistant *Salmonella* Typhimurium isolates (V57, V58 and V60). However, AS-PCR-RFLP revealed that all 3 isolates had double point-mutations in their *gyrA* gene at Ser-83 and Asp-87. Nucleotide sequencing of the fragments, spanning the “Quinolone Resistance Determining Region” (QRDR) showed a substitution in the codon TCC (Ser) at position to 83 TTC (Phe) and in the codon GAC (Asp) at position 87 to AAC (Asn).

## Discussion

To date very little data has been published on antimicrobial resistance among non-typhoidal *Salmonella* serovars from Vietnam [19]. A phenotypic resistance study is the first step of such an antimicrobial resistance investigation. The data from the present study indicated a high rate of antimicrobial resistance among Vietnamese *Salmonella* isolates. More than half of the isolates showed resistance to at least one antibiotic. The resistance percentages to chloramphenicol, streptomycin, ampicillin, sulphonamides, and tetracycline found in the present study were comparable to those found in other countries [20], [1], [21] and can therefore be considered a worldwide problem. The high rate of resistance of the Vietnamese isolates against aminoglycosides and trimethoprim differs from the low rate of resistance against these antimicrobials among *Salmonellae* isolated in 10 European countries [21]. An explanation for this observation may be the increasing and inappropriate use of antibiotics during the last ten years in Vietnam especially in the intensive animal husbandry in which antibiotics are being used on a large scale for prophylaxis, as growth enhancer, and for therapy. In 2002, gentamicin and trimethoprim, for example, were used frequently in animal husbandry in Vietnam [22].

The prevalence of integrons found in *Salmonella* varies from country to country and depends on the origin of the isolates. If both human and animal *Salmonella* isolates are included, 28%, 20%, and 16% of the Vietnamese, English, and Dutch non-typhoid *Salmonellae* isolates, respectively, were found to carry class 1 integrons as demonstrated in this study and in the literature [7], [23]. Among the 9 profiles of class 1 integrons found, gene cassettes encoding resistance to aminoglycosides (*aadA1*, *aadA2*, and *aadA5*), β-lactams (*bla*_PSE-1_, *bla*_OXA-30_) and trimethoprim (*dfrA1*, *dfrA12*, *and dfrA17*) were frequently detected. The data of the genotypic and phenotypic resistance assays in the present study indicated that apparently there is a relationship between the use of these antimicrobials in the last decades in human medicine and in the veterinary sector in Vietnam. In addition, the *sat* gene encoding resistance to streptothricin was also found.

An important observation in the current study was the high prevalence of class 1 integrons, especially in *S.* Typhimurium pt 90. In this study an integron of about 1.95 kb with the *aadA2*, *orfF* and *dfrA12*genes was the predominant integron profile detected in *S.* Typhimurium pt 90, *S.* Schwarzengrund, *S.* Anatum and *S.* Derby isolates. This type of class 1 integron has also been detected in *S.* Cholerasuis in Taiwan [24], and *S.* Gallinarum in Korea [25], in *S.* Schwarzengrund from catfish and squid roll imported from Thailand and Taiwan, respectively, to the United States [26]. During the same period, in European countries and the United States, human and animal *S.* Typhimurium strains (especially DT104) with the two integrons of the *aadA2* and *bla*_PSE-1_ genes were the most prevalent type [27], [28], [29]. Thus, different types of integrons can be dominant in different geographic regions. Also in this study, *S.* Typhimurium pt 90 isolates carried integrons and antibiotic resistance determinants against 7 to 8 different antimicrobials that could be transferred to *S.* Enteritidis and to *E. coli*. *S.* Typhimurium pt 90 is the most common phage type in Vietnam [30]. This suggests that *S.* Typhimurium pt 90 may play an important role in the spread of class 1 integrons and antimicrobial resistance determinants among Enterobacteriaceae in this country. Remarkably, 3 integrons (with amplicons of 1.7 kb, 1.95 kb and 2.0 kb) were detected in a single isolate (V57). This isolate was cultured from a serious case of human salmonellosis in Ho Chi Minh City. The isolate is classified as *Salmonella* Typhimurium U320 in the English phage typing system. The isolate was resistant to 13 antimicrobials including the fluoroquinolones. The spread of such *Salmonella* strains is hazardous and should be controlled.

Resistance to nalidixic acid (35%) and decreased susceptibility to fluoroquinolones (15%) of the isolates in the present study were even higher than in other Asian countries [1], [19]. Resistance to relatively new antimicrobials like norfloxacin and ciprofloxacin was found only among the human *Salmonella* isolates. This is of particular concern because ciprofloxacin is the drug of choice for the treatment of invasive human *Salmonella* infections. Mutations leading to substitutions at amino acid 83 and 87 of the QRDR may be in part responsible for the high level of resistance to fluoroquinolones (MIC norfloxacin = 32–64 µg/ml) among the 3 MDR resistant *Salmonella* Typhimurium isolates. These mutations lead to the substitution of Ser for Phe and Asp for Asn, at positions 83 and 87, respectively. This is the first report on mutations in two codons in *gyrA* of Vietnamese *Salmonellae*. Similar mutations have been found in *S.* Cholerasuis isolated from pigs in Taiwan [24]. It is important to note that the acquisition of fluoroquinolone resistance in *Salmonella* requires the stepwise accumulation of *gyrA* mutations or the overexpression of efflux pumps [31]. A single mutation in *gyrA* of *Salmonella* can be sufficient to cause high-level resistance to nalidixic acid but additional mutations are required to attain high-levels of resistance to fluoroquinolones [32]. Mutations in two codons are rarely found among field isolates of *Salmonella* while mutations at either Ser83 and or Asp87 are very commonly observed [33]. Resistance to antimicrobials in human *Salmonella* isolates can be the result of antibiotic misuse in human medicine: in Vietnam patients can easily buy antimicrobial drugs in any pharmacy without a prescription and stop treatment at any time. In addition, abuse of antibiotics in veterinary practice may have an important influence on selection of fluoroquinolone-resistant *Salmonella* isolates.

Unlike plasmid-mediated resistance, which may disappear in the absence of selective pressure, chromosomally mediated resistance is often maintained. Many MDR *Salmonella* isolates in this study contained SGI1 or one of its variants. This study documents the presence of a class 1 integron and SGI1-C in serovar Tallahassee. Thus worldwide, class 1 integrons and SGI1 are more and more recognized as significant determinants of multiple drug resistance in an increasing number of *Salmonella* serovars.

*S.* Emek is one of the dominant serovars found in poultry in Vietnam [30]. An important finding of our study was SGI1-J3 in a *S.* Emek isolate. This SGI1 has previously been described for *S.* Virchow and is integrated into SGI gene S023 instead of at the usual position between genes S027–S044 [16]. To the best of our knowledge, this is the second report on the insertion of a SGI1 at this position, however here it is in a different *Salmonella* serovar. SGI-J3 is closely related to SGI-J2 which was described for *S.* Emek isolates, the main difference being the presence of a large part of the *tni* module of Tn*5058* in SGI-J3 [16], [17]. Levings et al. [17] chose to rename SGI1-J as SGI2, but we adhere to the nomenclature of Doublet et al [16]. Whether the *tni* module of Tn*5058* was acquired by SGI1-J3 or lost by SGI-J2 is a matter of speculation, but the *S.* Emek described in this study may form a missing link. Its integron is identical to that of *S.* Virchow, whereas SGI1-J2 is present in the same serovar, but lacks the *tni* module.

In the present study, SGI1 was detected as circular extrachromosomal DNA in *S.* Typhimurium DT104 isolates but not in other SGI1 carrying serovars. This suggests that *S.* Typhimurium DT104 may play a key role in the spread of SGI1 among *Salmonella* serovars because the extrachromosomal circular intermediate of SGI1 can be transferred in the presence of a helper plasmid providing the mating apparatus as described previously [18].

In summary, high rates of multidrug resistance and of the presence of integrons found among the *Salmonella* isolates in this study suggests that legislation to enforce a more prudent use of antibiotics in both human and veterinary medicine should be implemented by the authorities in Vietnam. The association of antimicrobial resistance determinants with transferable elements may promote the rapid dissemination of antibiotic resistance among Enterobacteriaceae. The diversity of transferable and novel multiresistance determinants observed in *Salmonella* serovars indicates that international co-operation is needed in order to limit the emergence and the spread of MDR *Salmonella* isolates, especially in the context of increased international travel and trade in food products of animal origin.

## Materials and Methods

### Isolates

A total of 297 epidemiologically unrelated isolates from Vietnam was investigated. The isolates originated from humans (n = 56), cattle (n = 63), pigs (n = 111), and poultry (n = 67). All animal isolates were collected during the year 2004. The animal isolates were cultured from faeces, carcasses and meat. Faecal samples from healthy animals were taken at slaughterhouses (78%) and from healthy or sick animals on farms (12%) as previously described [30]. The animal samples came from different flocks or herds. If more than one sample from a slaughterhouse, farm, market or supermarket was *Salmonella* positive, only one isolate was randomly chosen and included in this study. The 56 clinical human isolates of unrelated patients with diarrhoea and fever were obtained from five provincial hospitals and two Pasteur Institutes in Vietnam. These isolates had been isolated during the year 2004. The methods used for the isolation and identification of the isolates have been described [30]. The isolates included in the present study belonged to 38 serovars of *Salmonella*. *S.* Typhimurium, *S.* Anatum, *S.* Weltevreden, *S.* Emek and *S.* Rissen were the most prevalent serovars. *S.* Typhimurium phage type 90 (in the Dutch phage typing system), which has no recognized phage type in the English phage typing system, was predominant among the *S.* Typhimurium isolates [34].

### Antimicrobial Susceptibility Testing

The antimicrobial susceptibility of the isolates was determined according to the guidelines of the Clinical and Laboratory Standards Institute (CLSI) [35]. Agar diffusion assays were performed on Muller-Hinton agar and with disks containing 15 different antimicrobial agents (Oxoid, UK). The antimicrobials tested (Table 1) were ampicillin 10 µg (A), amoxicillin/clavulanic acid 30/15 µg (Ac), cefalothin 30 µg (Ce), ceftazidime 30 µg (Cf), chloramphenicol 30 µg (C), ciprofloxacin 5 µg (Ci), colistin 10 µg (Co), gentamicin 10 µg (G), kanamycin 30 µg (K), nalidixic acid 30 µg (Na), norfloxacin 10 µg (No), streptomycin 10 µg (S), tetracycline 30 µg (T), trimethoprim 5 µg (Tp), and sulphonamides 300 µg (Su). The interpretive categories susceptible, intermediate or resistant were used according to the CLSI guidelines [36] except for colistin where the zone criteria of ≤11 (resistant) and ≥14mm (susceptible) were used [37]. *Escherichia coli* ATCC 25922 and *E. coli* ATCC 35218 were used as quality control organisms.

### Detection of Class 1 Integrons

All isolates were tested for the presence of class 1 integrons. The presence of integrons was determined by PCR amplification of the class 1 integrase specific *int1* gene [38]. Template DNA was obtained by the whole cell boiled lysate method [39]. Integron gene cassettes were detected by PCR using the 5′-CS and 3′-CS primer set [39]. CS-PCR products were separated in 0.7% agarose gels for at least 3 hours at 100 V and visualized under UV-light after staining with ethidium bromide. Since the 3′-CS fragment of class 1 integrons is not always as conserved as its name indicates [40], an integrase-positive isolate does not always yield an amplicon in the CS-PCR. If this was the case, an integrase-inverted PCR was used to characterise the gene cassettes.

Briefly, 1 µg of genomic DNA of the isolate was cleaved with the restriction endonuclease *Sph*I. The fragments were ligated and subjected to PCR using int-OUT and CS-F primers. Since the smallest size for a gene cassette inserted into an integron is about 400 bp only fragments larger than 500 bp generated from the inverted PCR were sequenced.

### Characterization of Integrons

CS-PCR products with the same size were purified using the QiaQuick PCR purification kit (Qiagen, Germany) and analysed by restriction fragment length polymorphism (RFLP). The amplicons were digested with at least two different restriction endonucleases and the order and arrangement of the gene cassettes was considered identical if they showed the same RFLP patterns. The restriction endonucleases used were *Hpa*II, *Hinc*II, *Bcl*I, *Nci*I, and *Eco*RI.

### Nucleotide Sequencing of Gene Cassettes

One representative of each RFLP type was randomly chosen for nucleotide sequencing. For isolates with a unique integron, purified CS-PCR products were cloned in the pGEM-T easy Vector (Promega, USA). Colonies carrying the inserted fragment were picked from Luria Bertani plates containing ampicillin (100 µg/ml), 40 µl (100 mM) IPTG and 40 µl (2%) X-Gal. The inserted fragments were obtained by PCR, using T7 and SP6 primers under the same conditions as for the CS-PCR. The amplification products were purified and sequenced. The T7 and SP6 primers were used for sequencing both ends of the different amplicons under study. In addition, for the 2000 bp amplicon obtained by CS-PCR, an internal primer was used to continue the sequencing until the resistance genes in each amplicon were identified. For isolates carrying two integrons which only differed about 50 bp in size, CS-PCR products were also cloned in the pGEM-T easy Vector. The two plasmids with different inserts were selected based on restriction enzyme (*Eco*RI or *Hpa*II) analysis and used for sequencing. Dideoxy sequencing was performed on an ABI 3730 Sequencer. DNA sequences were analysed with the Clone Manager Suite and by consulting the GenBank database via the BLAST network service. The nucleotide sequences of the gene cassettes have been deposited in the GenBank database under the accession numbers shown in Table 2.

### Bacterial Conjugation

A conjugation experiment was performed as described [41] to determine whether the integrons of the *Salmonella* isolates were on conjugative DNA elements and resistance determinants could be transferred to another *Salmonella* serovar or to another bacterial species (*E. coli*). Rifampicin-resistant (Rif^R^) and sulfamethoxazole-susceptible (Sul^S^) *E. coli* K12 was used as recipient. All 83 integron carrying *Salmonella* isolates were used as donor strains. In addition, a plasmid-free susceptible *S.* Enteritidis isolate, which was made resistant to rifampicin, was also used as recipient. In this second conjugation experiment, only *Salmonella* isolates which could transfer their resistance determinants to *E. coli* in the first conjugation experiment were used as donors. Both donor (*int*-positive sulfamethoxazole-resistant *Salmonella* isolates) and acceptor bacteria were cultured in LB broth until an OD_660_ = 0.5–0.6 was reached. The mating process in which donor (sulfamethoxazole-resistant isolates) and recipient were present in a 1∶9 ratio (v/v) was performed in LB broth. Incubation took place overnight in a water-bath at 37°C. Transconjugants were selected by plating 50 µl of the mating culture on MacConkey (Oxoid, UK) agar plates containing both rifampicin (50 µg/ml) and sulfamethoxazole (512 µg/ml). Colonies were selected based on their resistance to both antimicrobials and purified by subculture on MacConkey agar containing antibiotics and then nutrient agar (NA, Oxoid, UK) without antibiotics. The transconjugants were tested for their biochemical characteristics, using the API 20E system (bioMérieux, France) and the *Salmonella* transconjugants were serotyped using antisera (Staten Serum Institute, Denmark) against antigens of *S.* Enteritidis. The transconjugants were tested as described above for their susceptibility patterns and the presence of class 1 integrons.

### Detection of Salmonella Genomic Island 1 and Its Variants

Integron-positive isolates (n = 12) from various *Salmonella* serovars were selected for analysis of the presence of SGI1 on the basis of their integron profiles and antibiotic resistance patterns. First, the isolates were examined for the presence of the left and right junction of SGI1 by PCR. Then the order of the antibiotic resistance gene cluster was tested as described [12]. Template DNA was prepared using the High Pure PCR Preparation Kit (Roche, Germany). The primers used for amplification of the left and right junctions of SGI1 and the linking sequences in the antibiotic resistance gene cluster were previously described [12], [14]. Primers 5′-AATTATCTCGTTCTGCATCC-3′ and 5′-CTCTGTTGTCGCAAGAAATG-3′ were used to demonstrate integration into SGI1 gene S023. The PCRs were carried out in a total volume of 25 µl volumes containing 2.5 µl of 10× PCR buffer (HT Biotechnology, England), 0.5 µl 10× deoxynucleotide triphosphate mix (2mM each), 50 pmol of each primer, 1.25 U Taq DNA polymerase, and 1 µl of template DNA. To amplify fragments larger than 3.5 kb, Taq Plus polymerase was used instead of Taq DNA polymerase (HT Biotechnology, England). Thermal cycling conditions consisted of a hot start cycle of 94°C for 3 min, followed by 35 cycles with: 1 min at 94°C, 1 min at 50 to 65°C (depending on the primers), 1 to 5 min at 72°C (depending on the expected amplicon size) and a final step at 72°C for 10 min. The expected sizes of the PCR products were based on nucleotide sequences present in GenBank under accession number AF261825. *S.* Typhimurium N216 carrying SGI1 and *S.* Albany N107 containing SGI1-F [23] were included as positive controls. Amplification products of *S.* Emek V14 generated by PCR mapping were partially sequenced (Baseclear, The Netherlands).

### Determination of the Circular Extrachromosomal form of SGI1

From isolates harbouring SGI1 or its variant types, 8 representative isolates carrying SGI1 or one of its variants were randomly chosen and examined for the presence of the circular extrachromosomal form of SGI1 by PCR (SGIc-PCR). The PCR was performed using primers oriented towards the left and right chromosomal SGI1 junctions and plasmid DNA extracted with the Qiagen plasmid midi kit (Qiagen, Germany) as template DNA [18]. The obtained PCR product was subsequently sequenced.

### Fluoroquinolone Resistance

Three fluoroquinolone (norfloxacin/ciprofloxacin)-resistant *Salmonella* Typhimurium isolates (V57, V58 and V60) were further studied with respect to the resistance mechanism involved. A PCR described by Paauw et al [42] was used to investigate whether the class 1 integron-associated- *qnrA* gene cassette was present. A *qnr*-carrying *Enterobacter cloacae* strain [42] was used as positive control. Since a mutation in the target enzyme for fluoroquinolones, GyrA, is regularly found in *Salmonellae* [8], [43], [44], [45], the 3 fluoroquinolone-resistant isolates were subjected to allele-specific PCR and RFLP analysis (AS-PCR-RFLP) as described [43] to detect mutations related to quinolone resistance in codons 81, 83 and 87 of the *gyrA* gene. The *gyrA* mutations were confirmed by nucleotide sequencing of the products generated by PCR using GyrA-F and GyrA-R as primers [43].
